# Altered neutrophil-to-lymphocyte ratio in patients with non-affective first episode psychosis and its relationship with symptom severity and cognitive impairment

**DOI:** 10.1038/s41598-023-37846-y

**Published:** 2023-07-15

**Authors:** Kwan Keung Leung, Yip Chau Wong, Ka Sin Shea, Sheung Chun Chan, Wing Chung Chang, Yi Man Flora Mo, Sau Man Sandra Chan

**Affiliations:** 1 Department of Psychiatry, Tai Po Hospital, G30, Ground Floor, Multicentre, Tai Po Hospital, Tai Po, New Territories, Hong Kong, SAR China; 2 Department of Psychiatry, Alice Ho Miu Ling Nethersole Hospital, Hong Kong, China; 3 Department of Psychiatry, Faculty of Medicine, The Chinese University of Hong Kong, Hong Kong, China; 4 Department of Psychiatry, Faculty of Medicine, The University of Hong Kong, Hong Kong, China

**Keywords:** Inflammation, Predictive markers

## Abstract

Signatures of immune dysregulation as clinical biomarker for psychosis have remained unclear. We aimed to compare the Neutrophil-to-lymphocyte ratio (NLR) of patients with acute non-affective first-episode psychosis (FEP) with healthy controls after accounting for emotional states. We also explored the associations of NLR with symptom severity, onset profile and cognitive functions. The NLR was enumerated from complete blood count taken within a week of assessment. All FEP patients were rated on the Positive and Negative Syndrome Scale (PANSS) and the Clinician Global Impression-Severity (CGI-S) with verbal memory and executive functions assessed with the Cambridge Neuropsychological Test Automated Battery. Prevailing emotional state was measured with Beck Depression Inventory-II and Beck Anxiety Inventory. Out of seventy-nine consecutive FEP patients presenting to the study site, twenty-seven subjects were eligible and recruited. Twenty-seven age-/sex-matched controls were recruited. FEP patients had an NLR of 1.886 over the controls after accounting for scores on emotional states. The NLR of FEP patients was positively associated with CGI-S scores, PANSS positive symptom, disorganization and excitation scores. There was no significant correlation between NLR with the duration of untreated psychosis and cognitive performances. These findings support using NLR as a clinical biomarker in FEP, purporting further prospective study to measure NLR changes in the course of treatment.

## Introduction

Inflammation and immune dysregulation have been implicated in the pathogenesis of major neuropsychiatric disorders, including mood or anxiety-related disorders^1^ and psychosis^2–5^. Alterations in circulating blood cytokine network were reported^6^. Abnormal immune cell counts have been reported in patients with schizophrenia and those with first-episode psychosis (FEP)^7–9^. Some studies revealed upregulation of pro-inflammatory cytokines^10^ and high sensitivity C-reactive protein (CRP)^11^ in psychosis. A recent meta-analysis also confirmed an imbalance of pro-inflammatory status and a reduction of anti-oxidant signals in patients with FEP compared with healthy controls^12^. Treatment with antipsychotics was shown to modulate the inflammatory status in patients with psychosis, although the effects were inconsistent across different psychotropics^13^. Other studies revealed differential immune status with levels of psychotic symptom severity^14^, and among antipsychotic responders or non-responders in FEP^15^. Symptoms aside, alterations of immune status were associated with abnormal cognitive functions in schizophrenia, as reported in a recent systematic review and meta-analysis. However, the associations found in these studies were weak, given the small effect size of the correlations reported^16,17^. Specific profiles of inflammatory biomarkers were reported to correlate with some cognitive domains in FEP patients^18^.

Routine serum assays of inflammatory biomarkers are often unavailable in psychiatric clinical settings. On the other hand, neutrophil-to-lymphocyte ratio (NLR) is a clinically accessible candidate biomarker easily enumerated from complete blood count (CBC) measured in routine clinical care. A ratio between the neutrophil and lymphocyte count conjugates innate and adaptive immunity. An increase in neutrophil levels could reflect an elevated systemic inflammatory status augmenting neutrophil-mediated killing with adaptive regulatory immunity in various diseases^19^. NLR has been reported as a prognostic indicator in disease entities like cancer^20^, metabolic syndrome^21^, sepsis^22^, and inflammatory bowel diseases^23^. A recent cross-sectional study revealed an increased NLR in more than 13,000 clinically diverse service users attending a regional psychiatric service in South London. Such transdiagnostic association in psychiatric service users was also influenced by age (positive correlation), psychotropics, and ethnicity (white over non-white) relative to the profiles of healthy controls (n > 3000) drawn from a National Health Survey^24^. Another recent meta-analysis pooling data from retrospective or cross-sectional studies showed an elevated NLR in patients with psychosis over healthy controls^25^. Emerging evidence supports a possible association of NLR with positive psychotic symptoms in schizophrenia^26^ and a positive association of higher NLR with the severity of psychotic symptoms in unmedicated patients with schizophrenia^27^. However, the results of these studies could not discriminate between clinically remitted patients and those who were acutely psychotic.

In summary, there are main knowledge gaps in the understanding of clinical correlates of NLR in psychosis. First, existing case-controlled studies in patients with psychosis did not account for the emotional states of the patients with psychosis even though stress and emotion have complex interplay with inflammatory activity^28^. Given some earlier reports suggesting the association of altered NLR in mood disorders^29^, the emotional state is a potential confounder to be considered in the analysis of NLR in psychosis. Further, the evidence from a retrospective review of a multi-ethnic clinical registry suggested the role of ethnicity in moderating the association of NLR and psychiatric disorder in a UK cohort^24^, in which data on Chinese subjects were lacking. It also remains unclear whether NLR is a state or trait marker in psychosis, and notably, its linkage to symptom severity and cognitive impairment has remained obscure. Many studies included patients with a relapsing course of schizophrenia, in which long illness duration and chronic psychotropic treatment might confound the clinical association of the inflammatory status^30^. Data were lacking in patients with FEP, a relatively homogenous clinical population in terms of duration of illness and exposure to psychotropics.

This case–control study primarily aimed to compare the NLR of ethnic Chinese patients with non-affective FEP and the age-/sex-matched healthy controls. It also aimed to explore the association of NLR with severity of psychotic symptoms and cognitive function after accounting for current emotional states.

## Methods

### Participants and settings

The current study was approved by the Chinese University of Hong Kong (CUHK) – NTEC Clinical Research Ethics Committee (CREC reference number: 2020.250). We recruited consecutive patients ages 18–50 years with non-affective FEP from two clinical settings: 1) hospitalized patients presenting to a regional tertiary psychiatric inpatient unit (Tai Po Hospital, TPH) serving the New Territories East Cluster under the public sector; 2) those referred for assessment at the FEP clinic of Alice Ho Miu Ling Nethersole Hospital (AHNH). Based on a similar study design comparing the blood parameters of FEP subjects and healthy control ^31^, the mean NLR assumed was 2.7 in FEP patients and 1.6 in control, and a pooled standard deviation of 1.9 and 0.6, respectively. Taking the probability of type I error (α) as 0.05 and power level (1-β) as 80%, a sample size of 27 per group would be needed to adequately test for difference in the NLR between the subject group and healthy control. The research team posted an advertisement at TPH/AHNH in the same study period, when adult healthy volunteers signed up and were enrolled as control participants only if their age and gender matched the recruited cases with non-affective FEP. Non-affective FEP patients and control participants with informed consent were recruited and assessed from November 2020 to July 2021. Inclusion and exclusion criteria were summarized in Supplementary Table 1. The exclusion criteria were infections, systemic diseases and metabolic problems, recent traumas or injuries, pregnancies, recent blood donations or transfusions, smoking, recent flu or COVID-19 vaccines, any medication or supplement use that potentially affects inflammatory status^25^. Non-affective FEP patients were defined as having the first inpatient psychiatric hospitalization or outpatient FEP consultation while meeting the diagnostic criteria for schizophrenia spectrum disorder in the Diagnostic and Statistical Manual of Mental Disorders-Fourth Edition (DSM-IV). All clinical diagnoses were confirmed with a validated Chinese-bilingual version of Structured Clinical Interview for DSM-IV Axis I Disorders (SCID-I) ^32^. All research was performed in accordance with relevant guidelines and regulations.

### Data collection and blood analysis

The demographic and clinical data of non-affective FEP subjects were collected from a direct interview supplemented with electronic clinical record review. The research interviews were conducted on FEP subjects within 1 week after presenting to the inpatient or outpatient unit. All blood samplings were done in the morning. The earliest available blood count values were used to minimize the potential effects of healthcare interventions. The same protocols for blood sampling and clinical interview were carried out for the healthy controls. The assessor was blinded to the blood parameters while performing all clinical interviews or assessments. All blood assays were processed in an accredited pathology laboratory in AHNH. The NLR of each participant was not analyzed until all clinical data were collected.

### Clinical and cognitive assessments

Psychiatric disorders were diagnosed with a validated Chinese-bilingual version of DSM-IV SCID-I^32^. Non-affective psychosis was defined as those diagnosed with schizophrenia, schizophreniform disorder, delusional disorder, or psychotic disorder not otherwise specified. The psychotic symptoms of all FEP patients were assessed using the PANSS with precise ratings^33,34^. PANSS positive, disorganization, negative, excitation, anxiety and depressive symptom scores were derived from previous factor-analytic study on FEP patients^35^. The emotional states were assessed with validated Chinese versions of BDI-II and BAI^36^ to record the prevailing mood symptoms in the past week. The neurocognitive assessment was carried out by a computerized system known as the CANTAB^37^. An earlier systematic review reported verbal memory and executive functions were the domains with the largest effect size in cognitive impairment of FEP^38^. Verbal Recognition Memory (VRM) tests, Spatial Working Memory (SMW) tests, and the One-Touch Stockings of Cambridge (OTS) tests were selected to assess verbal memory, working memory and executive functions. CGI-S was used to gauge the global clinical severity^39^.

### Statistical analysis

Power and sample size estimations were done using the computerized G-power analysis^40^. Statistical analyses were performed using the IBM Statistical Package for the Social Sciences for Windows Version 26.0 (IBM Corp., Armonk. NY, USA computer software). Descriptive statistics were used to summarize the demographic and clinical data. Continuous variables were expressed as means and standard deviations if the data were normally distributed, while median and interquartile ranges (IQR) if the data were not normally distributed. Categorical variables were presented as absolute numbers and proportions (percentages). A Kolmogorov–Smirnov test was used to examine each variable for normality. For comparing the sex- and age-matched subjects, continuous data were analyzed by paired t-test if the data were normally distributed. At the same time, Wilcoxon signed-rank test was used if the data were not normally distributed. Categorical data were analyzed by McNemar test (two categories only) and Marginal Homogeneity test (more than two categories). Spearman's rho correlation coefficient was used to investigate any significant relationship between two continuous variables. Generalized Estimating Equation (GEE) model was used in assessing the differences between the cases and controls by accounting for the working correlation matrix of putative confounders. The occupation status, years of education, BDI-II and BAI scores were included in the model for analyses. All tests were set with a significant level of *p* < 0.05.

## Results

### Characteristics of the sample

**Figure 1:**
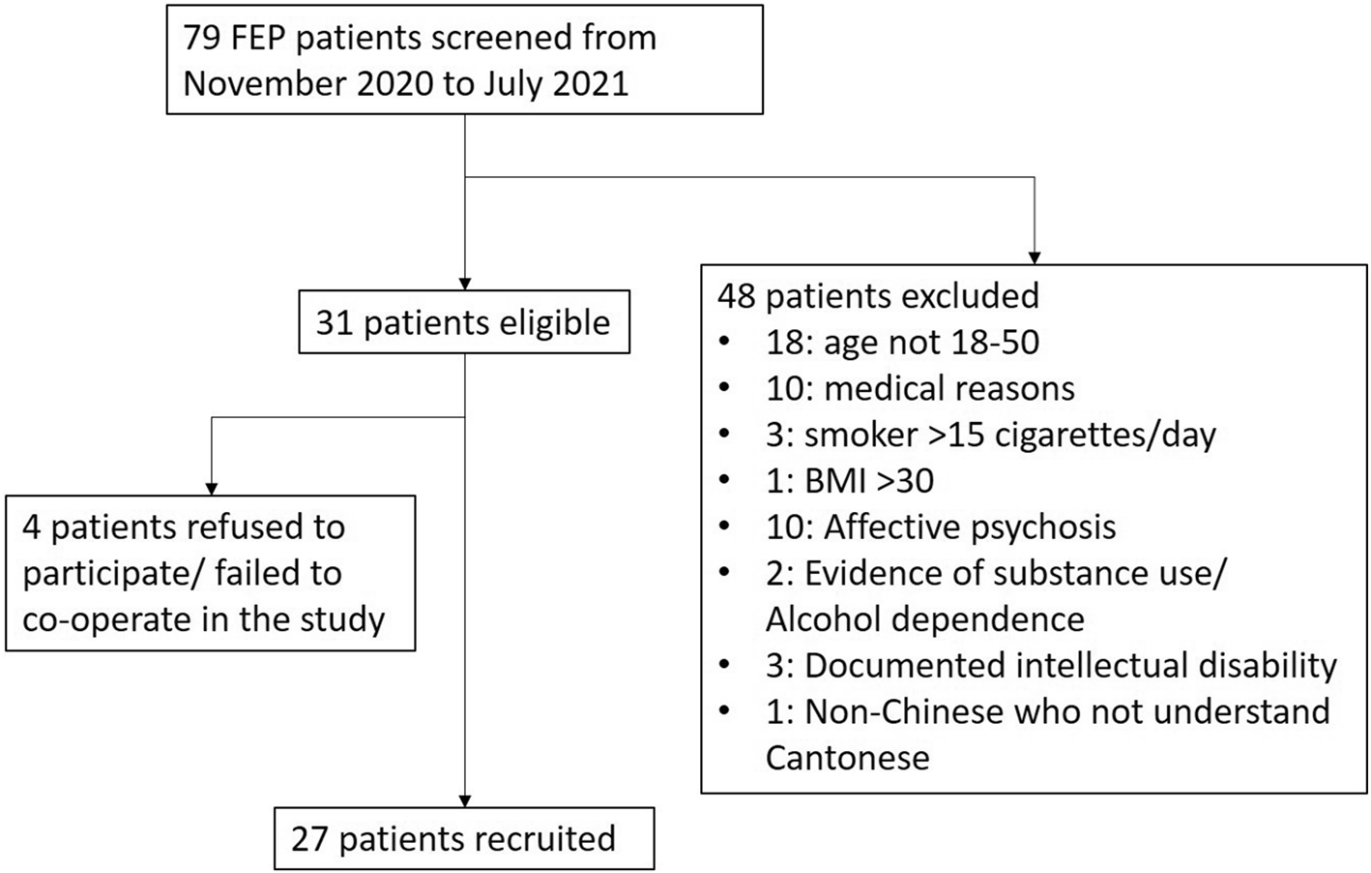
The study inclusion and exclusion process.

Consecutive FEP patients from November 2020 to July 2021 were screened for eligibility for the study as shown in Fig. 1. Seventy-nine patients were screened for eligibility. Forty-eight patients were excluded due to the conditions specified in the exclusion criteria. Among the thirty-one eligible patients, four refused to participate or failed to cooperate during the assessment. Twenty-seven patients were recruited, and twenty-six patients were hospitalized at recruitment. Age (+ /− 2 years)- and sex-matched healthy controls were enrolled in the same period through advertising. The demographic data and clinical parameters of the FEP patients and healthy controls are summarized in Table 1. The groups were comparable except in “years of education” and “occupation status”. The healthy controls had significantly higher years of education than the FEP patients, and all the healthy controls were employed at the time of the assessment. Twenty-seven FEP patients were respectively diagnosed with schizophrenia (n = 8), schizophreniform disorder (n = 4), psychosis not otherwise specified (NOS) (n = 12), and delusional disorder (n = 3).

**Table 1:** Demographic information and clinical parameters of both FEP patients and healthy control.

Variables of interest	Level	FEP	Control	*p* value
		(n = 27)	(n = 27)	
Demographics				
Female gender, n (%)		18 (66.67)	18 (66.67)	1
Age at entry, mean (SD)		30.56 (8.06)	31.15 (7.56)	0.065
Years of education, mean (SD)		15.8 (3.96)	20.1 (1.85)	* < 0.001
Occupation, n (%)	No	16 (59.26)	0 (0)	* < 0.001
	Yes	11 (40.74)	27 (100)	
Marital status, n (%)	Single	17 (62.96)	18 (66.67)	0.206
	Married	7 (25.93)	9 (33.33)	
	Divorced	3 (11.11)	0 (0)	
Number of cohabitants, mean (SD)		2.48 (1.42)	2.48 (1.37)	1
Religion, n (%)	No	21 (77.78)	22 (81.48)	1
	Yes	6 (22.22)	5 (18.52)	
Smoking status, n (%)	No	24 (88.89)	26 (96.3)	0.5
	Yes (< 15 cig/day)	3 (11.11)	1 (3.7)	
Body mass index, mean (SD)		22.11 (4.22)	20.73 (2.24)	0.171
Family history of psychosis, n (%)	No	22 (81.48)	26 (96.3)	0.125
	Yes	5 (18.52)	1 (3.7)	
Psychiatric diagnosis, n (%)				
Schizophrenia		8 (29.63)		
Schizophreniform disorder		4 (14.81)		
Psychosis NOS		12 (44.44)		
Delusional disorder		3 (11.11)		
Illness Characteristics				
Log DUP^a^, mean (SD)		0.837(0.646)		
Use of antipsychotics on presentation, n (%)	No	24 (88.89)		
	Yes (treatment ≤ 1 week)	3 (11.11)		
Symptom and clinical severity				
PANSS-positive symptom score, mean (SD)^b^		27.4 (6.39)		
PANSS-disorganization score, mean (SD)^b^		25 (5.3)		
PANSS-negative symptom score, mean (SD)^b^		27 (6.75)		
PANSS-excitation score, mean (SD)^b^		13.1 (4.12)		
PANSS-anxiety and depressive symptom score, mean (SD)^b^		13.3 (4.12)		
CGI-S score, mean (SD)		5.37 (1.08)		
Emotional states				
BDI-II, mean (SD)		13.1	4.67	* < 0.001
BAI, mean (SD)		14.8	4.19	* < 0.001

SD, standard deviation; Psychosis NOS = Psychosis not otherwise specified; DUP, duration of untreated psychosis; PANSS, Positive and Negative Syndrome Scale; CGI-S, Clinical Global Impression-Severity; BDI-II, Beck Depression Inventory-II; BAI, Beck Anxiety Inventory.

*Denotes *p* < 0.05.

^a^DUP was log-transformed for parametric analysis due to its skewed distribution.

^b^PANSS positive, disorganization, negative, excitation, anxiety and depressive symptom scores were derived on the basis of a previous factor-analytic study on FEP patients^35^.

Significant values are in bold.

### Comparison of NLR between FEP and control groups

Analyses of blood count parameters are summarized in Table 2. The FEP patients had a significantly higher total white cell count (*p* = 0.009) and neutrophil count (*p* < 0.001) but a significantly lower lymphocyte count (*p* = 0.002) than the healthy controls. There were no differences between the monocyte (*p* = 0.526) and platelet count (*p* = 0.469). The NLR was significantly higher in the FEP patients than the healthy controls (*p* < 0.001). All except three of the FEP patients were unmedicated. The medicated FEP patients had been prescribed the second-generation antipsychotics (2 with risperidone, 3- and 5-day treatment; 1 with olanzapine, 3-day treatment) within 1 week before hospitalization. A post-hoc analysis excluding the three medicated FEP patients found a significantly higher NLR in the FEP group than in the controls (*p* < 0.001). Excluding smokers in the analysis also yielded higher NLR in the FEP group than in the controls (*p* < 0.001).

**Table 2:** The blood count parameters of both FEP patients and healthy control.

Variable		FEP (n = 27)	Control (n = 27)	*p*-value
White cell count	Median (IQR)	6.7 (5.5–7.8)	5.4 (4.7–7.3)	*0.009
Neutrophil	Median (IQR)	4.6 (3.6–5.6)	3.2 (2.6–4.1)	* < 0.001
Lymphocyte	Median (IQR)	1.3 (1–1.9)	1.7 (1.4–2)	*0.002
Monocyte	median (IQR)	0.5 (0.4–0.6)	0.5 (0.4–0.5)	0.526
Platelet	Median (IQR)	242 (214–298)	255 (208–286)	0.469
NLR	Median (IQR)	3.6 (2.29–4.31)	1.78 (1.49–2.14)	* < 0.001
NLR (no antipsychotics, n = 24)	Median (IQR)	3.6 (2.28–4.28)	1.79 (1.55–2.16)	* < 0.001
NLR (non-smoker, n = 24)^	Median (IQR)	3.37 (2.28–4.22)	1.85 (1.47–2.09)	* < 0.001

IQR, interquartile range; NLR, Neutrophil-to-Lymphocyte Ratio.

*Denotes *p* < 0.05.

^1 pair of age- and sex-matched FEP and control subjects were both smokers thus n = 24.

Significant values are in bold.

Scores on both BDI-II and BAI were found to be significantly higher in the FEP patients than in the healthy controls (both *p* < 0.001), reflecting the differences in the mood state of FEP patients in the week before the assessment (Table 1). To adjust for the confounding effects of the mood state, a GEE model was used in assessing the differences in NLR between FEP patients and healthy controls by accounting for the working correlation matrix. Years of education and occupation status as potential confounding factors were also included in the model, as they were found to be different between FEP and control group. Table 3 showed that the FEP group, with NLR as the outcome, had a higher score (β = 1.886, confidence interval 1.076–2.695, *p* < 0.001) over the controls after adjusting for the scores on BDI-II/BAI, years of education and occupation. The analysis suggested that the FEP case would have an NLR value 1.886 times higher than the healthy control group after accounting for the differences in emotional state, years of education, and occupation status. Repeating the analyses excluding the three medicated FEP patients and their respective matched controls yielded a higher NLR in the FEP patients than in healthy controls (data not shown). As there was ample evidence that the body mass index (BMI) could affect inflammatory status, and hence NLR^41^, we also analyzed BMI as a potential covariate, even though the BMIs were comparable between the two groups. We found that the NLR of FEP subjects was still higher than the controls after accounting for BMIs (β = 1.641, confidence interval 1.144–2.137, *p* < 0.001) (Supplementary Table 2).

**Table 3:** A Generalized Estimating Equation (GEE) model to investigate the differences in NLR between FEP patients and healthy control with BDI-II scores, BAI scores, occupation, and years of education adjusted.

Model	Constant	β	SE-β	95% CI of β	*p* value
NLR	1.477				
Group: FEP case group (reference: control group)		**1.886**	**0.413**	**1.076 to 2.695**	* < 0.001
Occupation		− 0.289	0.399	− 1.071 to 0.494	0.47
Years of Education		0.022	0.046	− 0.068 to 0.111	0.634
BDI-II		− 0.035	0.021	− 0.076 to 0.007	0.103
BAI		0.025	0.014	− 0.002 to 0.052	0.066

Outcome: NLR.

β, beta-coefficient; CI, confidence interval; SE, standard error; BDI-II, Beck Depression Inventory-II; BAI, Beck Anxiety Inventory.

*denotes *p* < 0.05.

Significant values are in bold.

### Relationships of NLR with clinical and cognitive variables in FEP

In the correlation analyses, the NLR was found to be positively associated with PANSS-positive symptom score (ρ = 0.613, *p* < 0.001), PANSS-disorganization score (ρ = 0.566, *p* = 0.002), PANSS-excitation score (ρ = 0.628,* p* < 0.001) as well as PANSS-anxiety and depressive symptom score (ρ = 0.435, *p* = 0.023). It did not have a statistically significant correlation with the PANSS-negative symptom score (ρ = 0.272, *p* = 0.17). The NLR was also found to be positively associated with the CGI-S score (ρ = 0.644, *p* < 0.001) (Table 4). Further comparisons were made on the NLR of patients grouped by duration of untreated psychosis (DUP). No significant correlation was found in the NLR of FEP patients with their DUP (ρ = − 0.296, *p* = 0.134) (Table 4).

**Table 4:** The correlation analyses between NLR and clinical symptoms, onset profile as well as the CANTAB neurocognitive test results in FEP patients.

Variables of interest	Spearman's rho (ρ)	*p*-value
Clinical symptoms		
PANSS-positive symptom score ^b^	0.613	* < 0.001
PANSS-disorganization score ^b^	0.566	*0.002
PANSS-negative symptom score ^b^	0.272	*0.17
PANSS-excitation score ^b^	0.628	* < 0.001
PANSS-anxiety and depressive symptom score ^b^	0.435	*0.023
CGI-S score	0.644	* < 0.001
Onset profile		
Log DUP^a^	− 0.296	0.134
Neurocognitive tests		
Verbal memory		
VRM-FR	0.063	0.755
VRM-IR	− 0.156	0.438
VRM-DR	− 0.139	0.49
Executive function		
SWM-BE	0.145	0.471
SWE-S	− 0.035	0.861
OTS-PSFC	− 0.188	0.348
OTS-MLC	0.066	0.744

^a^DUP was log-transformed for parametric analysis due to its skewed distribution.

^b^PANSS positive, disorganization, negative, excitation, anxiety and depressive symptom scores were derived on the basis of a previous factor-analytic study on FEP patients^35^.

PANSS, Positive and Negative Syndrome Scale; CGI-S, Clinical Global Impression-Severity; DUP, duration of untreated psychosis; VRM, Verbal recognition memory test, -FR, free recall, -IR, immediate recognition, -DR, delayed recognition; SWM, Spatial working memory test, -BE, between errors, -S, strategy; OTS, One-touch stockings of Cambridge test, -PSFC, Number of problems solved on first choice, -MLC, median latency to first choice.

*Denotes *p* < 0.05.

Significant values are in bold.

Performances in all three components of VRM, including free recall (VRM-FR) (ρ = 0.063, *p* = 0.755), immediate recognition (VRM-IR) (ρ = − 0.156, *p* = 0.438), and delayed recognition (VRM-DR) (ρ = − 0.139, *p* = 0.49) were not correlated with the NLR. The performances in SWM-BE (ρ = 0.145, *p* = 0.471) and SWM-S (ρ = − 0.035, *p* = 0.861) were not correlated with the NLR. For the OTS, no significant correlations were found between the performances in both OTS-PFSC (ρ = − 0.188, *p* = 0.348) and OTS-MLC (ρ = 0.066, *p* = 0.744) with the NLR in the FEP patients (Table 4). No significant correlations were found when correlation analyses were performed between DUP and the scores on the CANTAB sub-tests. (Data not shown).

## Discussion

Our study is the first to report the clinical correlations of NLR in non-affective FEP presenting to a tertiary care setting in Hong Kong SAR, and was among the few clinical studies that examined the clinical correlations of NLR in psychosis with the inclusion of ethnic Chinese. NLR was significantly higher in non-affective FEP patients than in age-/sex-matched healthy controls after accounting for the emotional state, education, and employment status. Our findings were consistent with the results reported in the meta-analysis, which reported an increased NLR of 0.65 in a pooled sample of relapsed patients with schizophrenia than in healthy controls, and an increased NLR of 0.52 in the pooled FEP subgroup patients than in healthy controls^25^. A higher total white cell count and neutrophil count but a lower lymphocyte count were observed in the FEP patients in our study. It is worth noting that lymphocyte count comparisons varied across different studies^31,42–44^, and the mixed findings might reflect the complex relationships between lymphocyte level and clinical status, such as symptom severity, illness chronicity, and treatment status, as shown in an earlier meta-analysis^45^. An increase in neutrophil counts and a decrease in lymphocyte counts in other diseases might reflect an overall enhanced inflammatory intensity and an impaired immune system, respectively^46^. A possible underlying mechanistic cellular-based involvement in the predisposition to inflammatory states has been fueled but a recent study which reported an increased blood neutrophil extracellular traps (NETs) in patients with early schizophrenia compared with controls irrespective of antipsychotic treatment. An early stress with history of childhood maltreatment also linked to patients with increased NETs^47^.

Regarding the possible effect of drug treatment on NLR, other reports demonstrated years of antipsychotic treatment could reduce NLR^26^. The correlations between NLR and the different psychotic symptom severity could be altered by antipsychotic treatment^27^. Nonetheless, we still found a significantly higher NLR of the drug-naïve FEP patients than the healthy controls after excluding the three FEP subjects with prior antipsychotic treatment.

Existing literature often overlooked the confounding effects of the prevailing emotional state on inflammatory status. For instance, anxiety-related states^48^ or sadness^49^ might influence inflammatory status. The FEP patients in this study had more intense depressive and anxious states as measured with BDI-II and BAI, probably secondary to the underlying active psychotic symptoms and other social stressors. Interestingly, some published studies alluded to the effects of socioeconomic status, including education and income on inflammatory states, although there has not been any report on their direct association with NLR^50,51^. The correlations between the occupation status or years of education with BDI-II or BAI scores found no significant correlations (data not shown), with the variance inflation factor computed with either BDI-II or BAI scores toward occupation status and years of education to be 1.13, suggesting a low chance of multicollinearity between the variables in our data. Using a GEE model, a higher NLR in the FEP patients remained statistically significant when the emotional states, years of education, and occupation status were accounted for, implicating an independent association of higher NLR with FEP in an active psychotic state.

A higher NLR might allude to unfavorable prognosis in various medical conditions^21,22,52–54^. Some evidence suggests that inflammation is associated with the risk of suicide, at least in adolescents^55^. Some recent evidence supported the association of higher NLR with the severity of psychotic state in patients with schizophrenia^26,27^. Amongst the non-affective FEP patients (schizophrenia spectrum disorder) in this case–control study, we found positive correlations between symptoms severity (reflected by the PANSS factors, as well as the CGI-S) with higher NLR, but no significant correlations between NLR and the duration of untreated psychosis (DUP). Our study revealed no significant correlation between NLR and PANSS-negative symptom score, parallel to findings reported in the existing review^56^. Overall it suggested the potential role of NLR as a biomarker of clinical severity in the acute episode of FEP patients.

Some animal studies have reported an association of increased inflammation with cognitive impairment^57^, while clinical studies have reported transdiagnostic associations of higher NLR with cognitive impairment in different psychiatric patients, including older adults^58–62^. Our study did not find a significant correlation between NLR and the selected tests on executive function and working memory in FEP patients. Intriguingly, the positive association between higher NLR and cognitive impairment was not merely neurotoxic. Some components of inflammatory cytokines could exert neuroprotective effects in maintaining certain cognitive performances^63^. Social stressors, which inevitably affect the hypothalamic-pituitary hormonal axis, have been implicated in impairing cognitive functions in FEP patients^64,65^. Considering the varying stress level that was not quantified in this study and the complex interplay of inflammatory status and cognitive performance, NLR on its own is not a sensitive marker to predict the level of cognitive performance during the active psychotic states in patients with FEP who had a relatively short duration of untreated psychosis.

We considered the possibility of other associative factors, including the DUP, that have not been examined in previous studies. While the association of long DUP with cognitive impairment is generally accepted in chronic schizophrenia^66^, a recent meta-analysis concluded the DUP among FEP was not significantly related to the performances of most cognitive domains^67^. We also reported that our FEP patients across different DUPs performed similarly and had no statistically significant correlations in all the tested cognitive performances. Given the small sample size in this case–control study design, it might remain a caveat in interpretation that these sub-group analyses might not reflect a true null association of NLR with DUP.

The pre-requisite for applying NLR into clinical practice is defining the normative values of age-/ gender-/ethnic-specific NLR in a healthy population. An earlier study suggested the mean normal NLR in a healthy adult population was 1.65^68^, comparable to our present study showing a median NLR of 1.78 in the healthy controls. A more recent investigation that retrieved data from a routine examination center in Western China suggested a reference NLR could be 0.88 to 4.0 in a healthy ethnic Chinese Han population^69^. However, these studies offered limited information on the normative value of NLR, which is known to vary with age ^70^ and ethnicity^24^.

Our study's findings have to be interpreted by considering the methodological limitations. Biased sex distribution (18 females and 9 males) could be a potential bias, yet our control samples were sex- and age-matched with the FEP participants. Other published data also revealed no sex differences in NLR^27^. Rather than using a convenience sample for controls, healthy household family members of the FEP subjects could be recruited to optimize the matching of cases and controls as they might share closer environment such as diet. Aside, this case–control study did not measure and account for potential confounding factors such as differences in circadian rhythm in subjects across different seasons. Such uncontrolled confounder might possibly be remedied by measuring blood morning cortisol level, which is often unavailable in daily clinical settings. The inevitably stringent selection criteria has thus resulted in the small sample size, compromising the power of subgroup analyses and the generalizability of the observed results. In this regard, further studies by adding comparisons with FEP patients having affective symptoms might serve to offer valuable information not only to increase sample size, but also allowing subgroup analysis between patients with and without affective symptoms. It would also be of interest to include other possible psychiatric co-morbidities in FEP patients to assess their possible interactive or additive effects on NLR, given the transdiagnostic associations of NLR.

The observations in the present study offered preliminary evidence in Hong Kong Chinese that NLR might be used to guide the assessment of acute exacerbation in non-affective FEP patients. Such clinical utility is especially promising when symptom reporting might be hampered by a lack of insight and often unsatisfactory therapeutic alliance in psychiatric care settings. Future studies should prospectively examine the NLR changes and clinical correlates in a broader spectrum of patients (in terms of chronicity and severity of illness) to elucidate the potential prognostic utility of NLR.

## Supplementary Information

Supplementary Tables.

## Data Availability

The data generated and used for this study are available from the corresponding author upon reasonable request, and may be requested via email, schan@cuhk.edu.hk.
