# Supplementary material for: Altered neutrophil-to-lymphocyte ratio in patients with non-affective first episode psychosis and its relationship with symptom severity and cognitive impairment
Source: Sci Rep. 2023 Jul 15;13:11453. doi: 10.1038/s41598-023-37846-y (PMC10349799; doi:10.1038/s41598-023-37846-y)
Supplement: Supplementary file 1 — Supplementary Tables. [file 41598_2023_37846_MOESM1_ESM.docx]

**Supplementary table 1:**

The inclusion and exclusion criteria for selecting FEP subjects and healthy control

|  | **Inclusion criteria** |
| --- | --- |
| All | i). Age 18-50 |
|  | ii). Ethnic Chinese who were Hong Kong residents. |
|  | iii). Able to understand spoken instructions in Cantonese and read written traditional Chinese. |
|  | **Exclusion criteria** |
| FEP | i). DSM-IV diagnosis of alcohol or substance use disorder, as well as documented intellectual disability |
|  | ii). evidence of affective episodes (depressive, manic, hypomanic, or mixed episode) or psychosis induced by other medical conditions; |
|  | iii). Obesity (Body mass index > 30kg/m^2^) or heavy smoker (>15 cigarettes/day) [1, 2]; |
|  | iv). Currently taking anti-hypertensive medications, corticosteroids, non-steroidal anti-inflammatory drugs, antibiotics, or other immunomodulatory agents; also regular use of vitamins or folate supplements [3-4] |
|  | v). Currently taking clozapine or lithium |
|  | vi). Systemic diseases including chronic obstructive pulmonary disease, diabetes, hypertension, heart diseases, malignancies, hematological diseases, hepatic dysfunction, renal dysfunction, or rheumatic diseases |
|  | vii). Acute or chronic infectious diseases |
|  | viii). Pregnancy |
|  | ix). Extremely outlying white blood cell counts (>11 x 10^9^ cells/L) that might indicate possible underlying infection |
|  | x). Recent trauma or surgical interventions in the past 6 months |
|  | xi). Blood donor or recipient in the past 6 months |
|  | xii). Inoculations with influenza virus or COVID-19 vaccines in the recent 1 month (Evidence of lymphocytes count returning to baseline within 10 days post-injection of mRNA COVID-19 vaccines [5], within 14 days post-injection of inactivated COVID-19 vaccines [6], as well as within 28-days post-injection of influenza virus vaccines) [7]. |
| Healthy control | i). All exclusion criteria for FEP |
|  | ii). Lifetime diagnosis of any mental disorders, as well as those with a history of any documented or known use of psychiatric services |
|  | iii). History of exposure to antipsychotics, antidepressants, or mood stabilizers. |

References

1. Kulaksizoglu B., Kulaksizoglu S. Relationship between neutrophil/lymphocyte ratio with oxidative stress and psychopathology in patients with Schizophrenia. *Neuropsychiatr Dis Treat.* **12**, 1999-2005 (2016).

2. Semiz M. *et al.* Elevated neutrophil/lymphocyte ratio in patients with Schizophrenia. *Psychiatr Danub* **26**(3), 220-225 (2014).

3. Roffman J.L. *et al*. A Randomized Multi-Center Investigation of Folate Plus B12 Supplementation in Schizophrenia. *JAMA Psychiatry*. **70**(5), 481-489 (2013).

4. Tabatabaeizadeh S.A. *et al*. High Dose Supplementation of Vitamin D Affects Measures of Systemic Inflammation: Reductions in High Sensitivity C-Reactive Protein Level and Neutrophil to Lymphocyte Ratio (NLR) Distribution. *J Cell Biochem.* **118**(12), 4317-4322 (2017).

5. Sahin U. *et al*. COVID-19 vaccine BNT162b1 elicits human antibody and T H 1 T cell responses. *Nature* **586**(7830), 594-599 (2020).

6. Xia S. *et al*. Effect of an Inactivated Vaccine Against SARS-CoV-2 on Safety and Immunogenicity Outcomes: Interim Analysis of 2 Randomized Clinical Trials. *JAMA* **324**(10), 951-960 (2020).

7. Faguet G.B. The effect of killed influenza virus vaccine on the kinetics of normal human lymphocytes. *J Infect Dis* **143**(2), 252-258 (1981).

**Supplementary table 2:**

A general linear model to investigate the differences in NLR between FEP patients and healthy control with Body Mass Index (BMI) adjusted. *denotes *p*<0.05. β = beta-coefficient; CI = confidence interval; SE = standard error

Outcome: NLR

| Model | Constant | β | SE-β | 95% CI of β | *p* value |
| --- | --- | --- | --- | --- | --- |
| **NLR** | 2.531 |  |  |  |  |
| **Group: FEP case group (reference: control group)** |  | **1.641** | **0.248** | **1.144 – 2.137** | ***<0.001** |
| BMI |  | -0.033 | 0.037 | -0.106 – 0.041 | 0.376 |
